# Dietary patterns and all-cause, cancer, and cardiovascular disease mortality in Japanese men and women: The Japan public health center-based prospective study

**DOI:** 10.1371/journal.pone.0174848

**Published:** 2017-04-26

**Authors:** Akiko Nanri, Tetsuya Mizoue, Taichi Shimazu, Junko Ishihara, Ribeka Takachi, Mitsuhiko Noda, Hiroyasu Iso, Shizuka Sasazuki, Norie Sawada, Shoichiro Tsugane, for the Japan Public Health Center-Based Prospective Study Group

**Affiliations:** 1 Department of Epidemiology and Prevention, Bureau of International Health Cooperation, National Center for Global Health and Medicine, Tokyo, Japan; 2 Prevention Division, Center for Public Health Sciences, National Cancer Center, Tokyo, Japan; 3 Department of Nutrition Management, Sagami Women’s University, Kanagawa, Japan; 4 Faculty of Human Life and Environment, Nara Women’s University, Nara, Japan; 5 Department of Diabetes Research, National Center for Global Health and Medicine, Tokyo, Japan; 6 Department of Endocrinology and Diabetes, Saitama Medical University, Saitama, Japan; 7 Department of Public Health, Division of Social and Environmental Medicine, Osaka University, Graduate School of Medicine, Osaka, Japan; 8 Epidemiology Division, Center for Public Health Sciences, National Cancer Center, Tokyo, Japan; 9 Center for Public Health Sciences, National Cancer Center, Tokyo, Japan; Leibniz Institute for Prvention Research and Epidemiology BIPS, GERMANY

## Abstract

**Objective:**

A meta-analysis showed an inverse association of a prudent/healthy dietary pattern with all-cause mortality and no association of a western/unhealthy dietary pattern. However, the association of distinctive dietary patterns of Japanese population with mortality remains unclear. We prospectively investigated the association between dietary patterns and all-cause, cancer, and cardiovascular disease mortality among Japanese adults.

**Methods:**

Participants were 36,737 men and 44,983 women aged 45–74 years who participated in the second survey of the Japan Public Health Center-based Prospective Study (1995–1998) and who had no history of serious disease. Dietary patterns were derived from principal component analysis of the consumption of 134 food and beverage items ascertained by a food frequency questionnaire. Hazard ratios of death from the second survey to December 2012 were estimated using cox proportional hazard regression analysis.

**Results:**

A prudent dietary pattern, which was characterized by high intake of vegetables, fruit, soy products, potatoes, seaweed, mushrooms, and fish, was significantly associated with decreased risk of all-cause and cardiovascular disease mortality. The multivariable-adjusted hazard ratios (95% confidence intervals) of all-cause and cardiovascular disease mortality for the highest versus lowest quartile of the prudent dietary pattern score were 0.82 (0.77 to 0.86) and 0.72 (0.64 to 0.79), respectively (*P* for trend <0.001 in both). A Westernized dietary pattern, characterized by high intake of meat, processed meat, bread, and dairy products, was also inversely associated with risk of all-cause, cancer, and cardiovascular disease mortality. A traditional Japanese dietary pattern was not associated with these risks.

**Conclusions:**

The prudent and Westernized dietary patterns were associated with a decreased risk of all-cause and cardiovascular disease mortality in Japanese adults.

## Introduction

Japanese life expectancy began to increase rapidly in the 1950s and has now become among the highest in the world [1]. Socioeconomic status, cultural background, and the Japanese diet might have contributed to Japanese population health [1]. Japanese food has a balanced nutritional profile, and the diet of the Japanese population has changed with economic development. For example, consumption of total fat (especially animal fat), animal protein, and calcium has increased with accompanying increases in consumption of meat and poultry and milk and dairy products [2]. The increase in the intake of these foods and nutrients after war achieved a peak in the 1970s [2]. The modern Japanese diet, which is somewhat westernized while maintaining aspects of the traditional diet, including regular consumption of fish and soy products, may have a beneficial effect on health.

A growing number of studies have examined the association of a priori and a posteriori dietary patterns, which integrate consumption of various foods or food groups, with mortality. For a priori dietary patterns, our study group recently reported that higher adherence to the Japanese Food Guide Spinning Top (balanced consumption of energy, grains, vegetables, fruits, meat, fish, eggs, soy products, and dairy products and limited consumption of confectionaries and alcoholic beverages), which was jointly developed by the Ministry of Health, Labour and Welfare and the Ministry of Agriculture, Forestry and Fisheries of Japan, was associated with decreased risk of mortality [3]. For a posteriori dietary patterns, the “prudent/healthy” dietary pattern, which is characterized by high intake of vegetables, fruits, fish, poultry, whole grains, and low-fat dairy products, has been associated with a decreased risk of all-cause and cardiovascular disease (CVD) mortality in a meta-analysis including 7 studies (6 in Western countries and 1 in Asia) for all-cause mortality and 6 studies (2 in Western countries and 4 in Asia) for CVD mortality [4]. However, it is not clear whether the distinctive dietary patterns of Japanese population is associated with mortality because only a few Japanese studies have examined the association of a posteriori dietary patterns with mortality from all causes (among the elderly) [5], CVD [6,7], and stomach cancer [8]. In addition, no study has examined the association of the Japanese diet with all-cause and major cause-specific mortality. Here, we prospectively investigated the association of major dietary patterns with the risk of all-cause, cancer, and CVD mortality in a large-scale population-based cohort study in Japan.

## Methods

### Study population

The Japan Public Health Center-based Prospective (JPHC) Study was launched in 1990 and 1993 for cohorts I and II, respectively [9]. Participants in cohort I were residents of five Japanese Public Health Center areas aged 40–59 years, and those in cohort II were residents of six other Japanese Public Health Center areas aged 40–69 years. The study sites are scattered across Japan but are mainly in rural areas. Baseline survey questionnaire was distributed to a total of 140,420 registered residents mostly by hand. Approximately 113,000 people returned the questionnaire, giving a response rate of 81%. The participants were informed of the objectives of the study, and those who completed the survey questionnaire were regarded as consenting to participation. The 5- and 10-year follow-up surveys (second survey and third survey, respectively) were conducted to update information on lifestyle habits and health conditions in 1995–1998 and 2000–2003, respectively. The present study used the second survey as the baseline.

Of the study population at baseline (n = 140,420), 102,695 participants (73%) responded to the second survey, including the diet-related portion. After exclusion of 1,065 participants who reported extreme total energy intake (sex-specific values outside of mean ± 3 standard deviations), 101,630 participants (47,408 men and 54,222 women) were included in analysis of dietary patterns. Of these, we excluded 12,867 participants who did not respond to the baseline survey and 7,043 participants who reported a history of severe disease, including cancer, cerebrovascular disease, myocardial infarction, chronic liver disease, and renal disease at the baseline or second surveys. Because severe disease probably influences dietary habit, we also used data of the baseline survey to carefully exclude participants with history of severe disease. Ultimately, 81,720 participants (36,737 men and 44,983 women) were enrolled.

### Ethics statement

This study was approved by the Institutional Review Board of the National Cancer Center of Japan and the Ethics Committee of the National Center for Global Health and Medicine, Japan.

### Dietary patterns

A food frequency questionnaire (FFQ) was used to assess the average intake of 147 food and beverage items over the previous year [10]. For most food items, participants were asked about consumption frequency and their usual portion size. The validity and reproducibility of the FFQ had already been established as reasonable [11–13].

Details of the identification of dietary patterns have been described elsewhere [14]. In short, we used 134 food and beverage items of the FFQ [excluding 11 items that correlated strongly with others and 2 items with no energy or nutrition (tap water and commercial water)]. Some foods within a food group with similar nutritional content or culinary use were combined, leaving 48 food group intakes. We performed principal component analysis based on intakes of these 48 food groups for men and women separately. The factors were rotated by orthogonal transformation (varimax rotation) to maintain uncorrelated factors and greater interpretability. We determined three dietary patterns based on eigenvalues, the scree test, and the interpretability of the factors in both men and women and designated them as follows: 1) a prudent dietary pattern, characterized by high intake of vegetables, fruit, soy products, potatoes, seaweed, mushrooms, and fish (including oily fish, seafood other than fish, and fish products); 2) a Westernized dietary pattern, characterized by high intake of meat (including pork and beef), processed meat, bread, dairy products, coffee, black tea, soft drinks, dressing, sauce, and mayonnaise; and 3) a traditional Japanese dietary pattern, characterized by high intake of salmon, salty fish, oily fish, seafood other than fish, and pickles [14]. The proportion of total variance explained by the three dietary patterns was 29.2% and 28.9% in men and women, respectively. The factor scores for each dietary pattern were calculated for each participant by summing intakes of food items weighted by their factor loadings. The scores were energy-adjusted using the residual method. The validity and reproducibility of the identified dietary patterns were acceptable [14]. When dietary patterns were extracted among the present analytic cohort (n = 81,720), similar dietary patterns emerged.

### Follow-up and outcome

The participants’ residency and vital status were followed up using the residential registry. Causes of death were confirmed via death certificates (with permission) and were defined according to the tenth revision of the International Classification of Diseases (ICD-10). The principal outcomes of this study were all-cause, cancer (ICD-10: C00-C97), and CVD mortality (ICD-10: I00-I99). CVD mortality was subdivided into mortality from heart disease (ICD-10: I20-I52) and cerebrovascular disease (ICD-10: I60-I69).

### Other variables

Information on height, body weight, medical history, smoking, physical activity, and other lifestyle factors was obtained via a self-administered questionnaire. Body mass index (BMI) was calculated as weight in kilograms divided by squared height in meters.

### Statistical analysis

Person-years of follow-up were calculated for each person starting from the date of response to the second survey questionnaire until either the date of death or December 31, 2012, whichever came first. Participants were divided into quartiles of factor score for each dietary pattern based on the separate distribution for men and women, and data for men and women were then combined. For baseline characteristics across each dietary pattern score, age-adjusted means and proportions were calculated using analysis of variance and logistic regression, respectively. Hazard ratios and 95% confidence intervals of mortality for quartiles of score for each dietary pattern were estimated using Cox proportional hazard regression analysis. The first model was adjusted for age (years, continuous), sex, and study area (11 areas), and the second model was further adjusted for BMI (<21.0, 21.0–22.9, 23.0–24.9, 25.0–26.9, or ≥27.0 kg/m^2^), smoking status (lifetime non-smoker, former smoker, or current smoker, with a consumption of either <20 or ≥20 cigarettes/day), total physical activity (quartile of metabolic equivalent task-hours/day), history of diabetes mellitus (yes or no), history of hypertension (yes or no), and total energy intake (kcal/day). An indicator variable for missing data was created for each covariate. We repeated the analysis after exclusion of deaths occurring in the first 3 years of follow-up. We also analysed the association between dietary pattern and all-cause, cancer, and CVD mortality by BMI (<25 kg/m^2^ or ≥25 kg/m^2^), hypertension (yes or no), sex, and age (<60 years old or ≥60 years old). An interaction term, created by multiplying dietary pattern score (quartile) and the above stratifying variables (dichotomous), was added to the model to assess statistical interactions. Two-sided *P* values less than 0.05 were regarded as statistically significant. All analyses were performed using Statistical Analysis System (SAS) version 9.3 (SAS Institute, Cary, NC, USA).

## Results

Characteristics of subjects according to quartile of dietary pattern score are shown in Table 1. The percentage of women was 55.0% in all diet categories because participants were divided into quartiles of dietary pattern score based on the separate distributions for men and women. Participants with higher score of the prudent dietary pattern were older, more likely to report histories of hypertension and diabetes, and less likely to be smokers than those with lower scores. Participants with higher score of the Westernized dietary pattern were younger and more likely to report lower levels of total physical activity. BMI was positively associated with the Westernized dietary pattern, whereas it was inversely associated with the traditional Japanese dietary pattern.

**Table 1:** Characteristics according to quartile (Q) of dietary pattern scores.

	Prudent pattern		Westernized pattern		Traditional Japanese pattern	
	Q1 (low)	Q4 (high)	Q1 (low)	Q4 (high)	Q1 (low)	Q4 (high)
**No. of participants**	20,429	20,430	20,429	20,430	20,429	20,430
**Age (year)**	55.9 ± 8.0	58.8 ± 7.9	60.1 ± 7.5	53.9 ± 7.4	57.9 ± 8.4	57.3 ± 7.6
**Sex (women, %)**	55.0	55.0	55.0	55.0	55.0	55.0
**Body mass index**^a^^,^^b^ **(kg/m**^**2**^**)**	23.5 ± 0.02	23.5 ± 0.02	23.4 ± 0.02	23.6 ± 0.02	23.9 ± 0.02	23.3 ± 0.02
**Current smoker**^a^^,^^b^ **(%)**	30.8	18.3	24.5	23.7	21.1	27.0
**Total physical activity**^a^^,^^b^ **(MET-h/day)**	33.1 ± 0.05	33.2 ± 0.05	33.7 ± 0.05	32.5 ± 0.05	33.2 ± 0.05	33.3 ± 0.05
**History of hypertension**^b^ **(%)**	15.9	17.8	17.8	15.9	17.6	17.8
**History of diabetes mellitus**^b^ **(%)**	4.0	6.1	5.0	4.3	4.8	4.2

Abbreviations: h, hour; MET, metabolic equivalent.

Data are mean ± standard error unless otherwise indicated.

^a^No. of participants with missing data were: BMI, n = 2,035; total physical activity, n = 14,361; smoking status, n = 3,731.

^b^Adjusted for age.

Daily food and nutrient intakes according to quartile of dietary pattern score are shown in Table 2. Participants in the highest quartile of the prudent dietary pattern score consumed more potatoes and starches, pulses, vegetables, fruits, mushrooms, algae, fish, and shellfish. They also consumed more polyunsaturated fatty acid, potassium, calcium, magnesium, iron, β carotene, vitamin B1, vitamin B2, vitamin B6, folate, vitamin C, dietary fiber, and salt. Participants with higher scores of the Westernized dietary pattern consumed more meats and milk and dairy products, and they consumed less salt. The high score of the Westernized dietary pattern was associated with high intake of total fat, especially saturated fatty acid and monounsaturated fatty acid. On the other hand, the traditional Japanese dietary pattern was inversely associated with fat intake. In addition, participants with higher scores of the traditional Japanese dietary pattern consumed more fish and shellfish, pickled vegetables, vitamin D, vitamin B12, and cholesterol.

**Table 2:** Daily food and nutrient intake^a^ according to quartile (Q) of dietary pattern scores.

	Prudent pattern		Westernized pattern		Traditional Japanese pattern	
	Q1 (low)	Q4 (high)	Q1 (low)	Q4 (high)	Q1 (low)	Q4 (high)
**Food intake**^b^ **(g)**						
**Cereals**	542 ± 1	513 ± 1	571 ± 1	494 ± 1	529 ± 1	528 ± 1
**Potatoes and starches**	16 ± 0	40 ± 0	29 ± 0	25 ± 0	28 ± 0	23 ± 0
**Pulses**	75 ± 1	103 ± 1	96 ± 1	77 ± 1	98 ± 1	79 ± 1
**Nuts and seeds**	1.4 ± 0.0	2.4 ± 0.0	1.7 ± 0.0	2.0 ± 0.0	1.7 ± 0.0	2.1 ± 0.0
**Vegetables**	134 ± 1	305 ± 1	236 ± 1	189 ± 1	226 ± 1	198 ± 1
** Pickled**	25 ± 0	45 ± 0	49 ± 0	21 ± 0	22 ± 0	46 ± 0
** Green and yellow**	59 ± 0	140 ± 0	100 ± 1	92 ± 1	102 ± 1	88 ± 1
**Fruits**	137 ± 1	285 ± 1	220 ± 1	201 ± 1	197 ± 1	200 ± 1
**Mushrooms**	5 ± 0	16 ± 0	10 ± 0	9 ± 0	9 ± 0	10 ± 0
**Algae**	7 ± 0	16 ± 0	12 ± 0	10 ± 0	12 ± 0	9 ± 0
**Fish and shellfish**	77 ± 0	94 ± 0	93 ± 0	80 ± 0	62 ± 0	109 ± 0
**Meats**	68 ± 0	46 ± 0	42 ± 0	73 ± 0	54 ± 0	67 ± 0
**Eggs**	30 ± 0	31 ± 0	30 ± 0	29 ± 0	30 ± 0	31 ± 0
**Milk and dairy products**	182 ± 1	194 ± 1	170 ± 1	192 ± 1	194 ± 1	173 ± 1
**Confectioneries**	13 ± 0	22 ± 0	14 ± 0	21 ± 0	18 ± 0	16 ± 0
**Alcoholic beverage**	249 ± 4	102 ± 4	148 ± 4	175 ± 4	151 ± 4	203 ± 4
**Non-alcoholic beverage**	723 ± 4	937 ± 4	739 ± 4	896 ± 4	864 ± 4	752 ± 4
**Nutrient intake**						
**Total energy intake (kcal/day)**	2148 ± 5	1824 ± 5	2051 ± 5	1997 ± 5	2022 ± 5	1939 ± 5
**Carbohydrate (% energy)**	52.2 ± 0.1	56.9 ± 0.1	56.4 ± 0.1	52.5 ± 0.1	54.9 ± 0.1	53.0 ± 0.1
**Protein (% energy)**	13.9 ± 0.0	14.6 ± 0.0	14.4 ± 0.0	14.2 ± 0.0	13.4 ± 0.0	15.1 ± 0.0
**Fat (% energy)**	25.2 ± 0.1	25.4 ± 0.1	23.0 ± 0.1	27.8 ± 0.1	25.8 ± 0.1	25.1 ± 0.1
** Saturated fatty acid (% energy)**	8.06 ± 0.02	7.21 ± 0.02	6.66 ± 0.02	8.67 ± 0.02	7.97 ± 0.02	7.39 ± 0.02
** Monounsaturated fatty acid (% energy)**	8.77 ± 0.02	8.56 ± 0.02	7.71 ± 0.02	9.73 ± 0.02	8.79 ± 0.02	8.73 ± 0.02
** Polyunsaturated fatty acid (% energy)**	5.26 ± 0.01	6.36 ± 0.01	5.67 ± 0.01	5.92 ± 0.01	5.87 ± 0.01	5.75 ± 0.01
**Sodium**^b^ **(mg)**	4075 ± 23	5144 ± 23	4887 ± 23	4327 ± 23	4356 ± 23	4804 ± 23
**Potassium**^b^ **(mg)**	2212 ± 4	3128 ± 4	2721 ± 5	2560 ± 5	2608 ± 5	2588 ± 5
**Calcium**^b^ **(mg)**	466 ± 2	587 ± 2	529 ± 2	505 ± 2	530 ± 2	495 ± 2
**Magnesium**^b^ **(mg)**	239 ± 0.3	304 ± 0.3	283 ± 0.4	257 ± 0.4	263 ± 0.4	270 ± 0.4
**Iron**^b^ **(mg)**	7.85 ± 0.01	9.95 ± 0.01	9.23 ± 0.02	8.47 ± 0.02	8.66 ± 0.02	8.97 ± 0.02
**Zinc**^b^ **(mg)**	8.05 ± 0.01	8.20 ± 0.01	8.20 ± 0.01	8.04 ± 0.01	7.84 ± 0.01	8.40 ± 0.01
**Retinol equivalent**^b^ **(μg)**	787 ± 4	938 ± 4	779 ± 4	943 ± 4	794 ± 4	969 ± 4
**β caroten equivalent**^b^ **(μg)**	2776 ± 21	6426 ± 21	4926 ± 23	3915 ± 23	5022 ± 23	3834 ± 23
**Vitamin D**^b^ **(μg)**	8.87 ± 0.04	10.11 ± 0.04	10.62 ± 0.04	8.56 ± 0.04	6.30 ± 0.04	12.61 ± 0.04
**Vitamin B1**^b^ **(mg)**	0.97 ± 0.00	1.09 ± 0.00	1.03 ± 0.00	1.01 ± 0.00	1.01 ± 0.00	1.03 ± 0.00
**Vitamin B2**^b^ **(mg)**	1.27 ± 0.00	1.50 ± 0.00	1.35 ± 0.00	1.39 ± 0.00	1.31 ± 0.00	1.41 ± 0.00
**Niacin**^b^ **(mg)**	17.8 ± 0.03	18.8 ± 0.03	17.8 ± 0.03	18.8 ± 0.03	16.8 ± 0.03	19.8 ± 0.03
**Vitamin B6**^b^ **(mg)**	1.31 ± 0.00	1.58 ± 0.00	1.50 ± 0.00	1.37 ± 0.00	1.32 ± 0.00	1.54 ± 0.00
**Vitamin B12**^b^ **(μg)**	8.03 ± 0.03	8.76 ± 0.03	8.74 ± 0.03	8.17 ± 0.03	6.04 ± 0.03	10.72 ± 0.03
**Folate**^b^ **(μg)**	303 ± 0.9	478 ± 0.9	405 ± 1.0	363 ± 1.0	376 ± 1.0	386 ± 1.0
**Vitamin C**^b^ **(mg)**	90 ± 0.4	183 ± 0.4	144 ± 0.5	124 ± 0.5	135 ± 0.5	126 ± 0.5
**Cholesterol**^b^ **(mg)**	286 ± 1.0	278 ± 1.0	270 ± 1.0	292 ± 1.0	254 ± 0.9	314 ± 0.9
**Dietary fiber**^b^ **(g)**	8.9 ± 0.03	16.1 ± 0.03	13.5 ± 0.03	11.0 ± 0.03	12.4 ± 0.03	11.6 ± 0.03
**Salt**^b^ **(g)**	10.3 ± 0.06	13.0 ± 0.06	12.3 ± 0.06	10.9 ± 0.06	11.0 ± 0.06	12.1 ± 0.06

Data are mean ± standard error.

^a^Adjusted for age.

^b^Adjusted for energy by using residual method.

During 1,212,808 person-years of follow-up (mean 14.8 years), we identified 11,012 all-cause deaths, 4,480 cancer deaths, and 2,813 CVD deaths, including 1,478 heart disease deaths and 1,096 cerebrovascular disease deaths. Hazard ratios of mortality according to quartile of each dietary pattern score are shown in Table 3. The prudent dietary pattern was significantly associated with decreased risk of all-cause, CVD, heart disease, and cerebrovascular disease mortality. The multivariable-adjusted hazard ratio (95% CI) of all-cause, CVD, heart disease, and cerebrovascular disease mortality for the highest quartile of the dietary pattern score was 0.82 (0.77 to 0.86), 0.72 (0.64 to 0.79), 0.75 (0.66 to 0.87), and 0.63 (0.53 to 0.75), respectively, compared to the lowest quartile. The risk of cancer mortality decreased with increasing score of the prudent dietary pattern after adjustment for age, sex, and area (*P* for trend <0.001). However, the association was attenuated after adjustment for covariates (*P* for trend = 0.21). The Westernized dietary pattern was also inversely associated with the risk of all-cause and CVD mortality, including heart disease and cerebrovascular disease mortality, although the magnitude of the decreased risk was not as large as that of the prudent dietary pattern. The inverse association between the Westernized dietary pattern and cancer mortality was observed even after adjustment for covariates. The traditional Japanese dietary pattern was not associated with the risk of any type of mortality. When we repeated the analysis after exclusion of deaths that occurred in the first 3 years of follow-up, we observed similar findings (data not shown).

**Table 3:** Hazard ratios (95% CI) for all-cause, cancer, and cardiovascular disease mortality according to quartile (Q) of dietary pattern scores.

	Q1 (low)	Q2	Q3	Q4 (high)	*P* trend^a^
**All-cause mortality**					
Prudent pattern					
Pearson-year of follow-up	302,606	305,023	305,089	300,090	
No. of deaths	2,970	2,480	2,531	3,031	
Age-, sex- and area-adjusted HR^b^ (95%CI)	1.00 (ref)	0.84 (0.80 to 0.89)	0.76 (0.72 to 0.80)	0.76 (0.72 to 0.80)	<0.001
Multivariate-adjusted HR^c^ (95% CI)	1.00 (ref)	0.89 (0.84 to 0.94)	0.81 (0.77 to 0.85)	0.82 (0.77 to 0.86)	<0.001
Westernized pattern					
Pearson-year of follow-up	302,495	305,860	304,406	300,046	
No. of deaths	3,885	2,895	2,319	1,913	
Age-, sex- and area-adjusted HR^b^ (95%CI)	1.00 (ref)	0.91 (0.87 to 0.96)	0.86 (0.81 to 0.90)	0.89 (0.83 to 0.94)	<0.001
Multivariate-adjusted HR^c^ (95% CI)	1.00 (ref)	0.93 (0.89 to 0.98)	0.88 (0.84 to 0.93)	0.91 (0.85 to 0.96)	<0.001
Traditional Japanese pattern					
Pearson-year of follow-up	295,318	301,277	306,490	309,723	
No. of deaths	3,006	2,425	2,502	3,079	
Age-, sex- and area-adjusted HR^b^ (95%CI)	1.00 (ref)	0.93 (0.88 to 0.99)	0.92 (0.86 to 0.97)	0.99 (0.93 to 1.05)	0.94
Multivariate-adjusted HR^c^ (95% CI)	1.00 (ref)	0.94 (0.89 to 0.999)	0.93 (0.87 to 0.99)	0.97 (0.91 to 1.03)	0.49
**Cancer mortality**					
Prudent pattern					
No. of deaths	1,133	1,019	1,071	1,257	
Age-, sex- and area-adjusted HR^b^ (95%CI)	1.00 (ref)	0.90 (0.83 to 0.98)	0.85 (0.78 to 0.92)	0.86 (0.79 to 0.93)	<0.001
Multivariate-adjusted HR^c^ (95% CI)	1.00 (ref)	0.94 (0.87 to 1.03)	0.91 (0.84 to 0.99)	0.95 (0.88 to 1.04)	0.21
Westernized pattern					
No. of deaths	1,514	1,146	967	853	
Age-, sex- and area-adjusted HR^b^ (95%CI)	1.00 (ref)	0.90 (0.83 to 0.97)	0.86 (0.79 to 0.93)	0.91 (0.83 to 0.99)	0.007
Multivariate-adjusted HR^c^ (95% CI)	1.00 (ref)	0.91 (0.84 to 0.98)	0.87 (0.80 to 0.94)	0.91 (0.83 to 1.001)	0.012
Traditional Japanese pattern					
No. of deaths	1,181	1,018	1,029	1,252	
Age-, sex- and area-adjusted HR^b^ (95%CI)	1.00 (ref)	0.99 (0.90 to 1.09)	0.97 (0.88 to 1.07)	1.06 (0.96 to 1.17)	0.20
Multivariate-adjusted HR^c^ (95% CI)	1.00 (ref)	0.99 (0.91 to 1.09)	0.98 (0.88 to 1.08)	1.04 (0.95 to 1.15)	0.41
**Cardiovascular disease mortality**					
Prudent pattern					
No. of deaths	800	632	649	732	
Age-, sex- and area-adjusted HR^b^ (95%CI)	1.00 (ref)	0.80 (0.72 to 0.89)	0.71 (0.64 to 0.79)	0.66 (0.60 to 0.74)	<0.001
Multivariate-adjusted HR^c^ (95% CI)	1.00 (ref)	0.85 (0.77 to 0.95)	0.77 (0.70 to 0.86)	0.72 (0.64 to 0.79)	<0.001
Westernized pattern					
No. of deaths	1,048	778	551	436	
Age-, sex- and area-adjusted HR^b^ (95%CI)	1.00 (ref)	0.94 (0.86 to 1.03)	0.81 (0.73 to 0.90)	0.84 (0.75 to 0.95)	<0.001
Multivariate-adjusted HR^c^ (95% CI)	1.00 (ref)	0.98 (0.89 to 1.07)	0.85 (0.76 to 0.94)	0.88 (0.78 to 0.99)	0.003
Traditional Japanese pattern					
No. of deaths	762	622	623	806	
Age-, sex- and area-adjusted HR^b^ (95%CI)	1.00 (ref)	0.91 (0.81 to 1.02)	0.85 (0.75 to 0.96)	0.94 (0.83 to 1.06)	0.35
Multivariate-adjusted HR^c^ (95% CI)	1.00 (ref)	0.94 (0.84 to 1.05)	0.88 (0.78 to 0.99)	0.93 (0.83 to 1.05)	0.23
**Heart disease mortality**					
Prudent pattern					
No. of deaths	426	299	342	411	
Age-, sex- and area-adjusted HR^b^ (95%CI)	1.00 (ref)	0.71 (0.61 to 0.83)	0.71 (0.62 to 0.82)	0.70 (0.61 to 0.80)	<0.001
Multivariate-adjusted HR^c^ (95% CI)	1.00 (ref)	0.77 (0.67 to 0.90)	0.78 (0.67 to 0.90)	0.75 (0.66 to 0.87)	<0.001
Westernized pattern					
No. of deaths	536	420	294	228	
Age-, sex- and area-adjusted HR^b^ (95%CI)	1.00 (ref)	0.99 (0.87 to 1.12)	0.83 (0.72 to 0.96)	0.84 (0.71 to 0.99)	0.006
Multivariate-adjusted HR^c^ (95% CI)	1.00 (ref)	1.03 (0.90 to 1.17)	0.88 (0.76 to 1.02)	0.88 (0.74 to 1.04)	0.042
Traditional Japanese pattern					
No. of deaths	429	312	353	384	
Age-, sex- and area-adjusted HR^b^ (95%CI)	1.00 (ref)	0.88 (0.75 to 1.04)	0.95 (0.80 to 1.12)	0.89 (0.75 to 1.06)	0.35
Multivariate-adjusted HR^c^ (95% CI)	1.00 (ref)	0.91 (0.77 to 1.07)	0.97 (0.83 to 1.15)	0.88 (0.74 to 1.04)	0.22
**Cerebrovascular disease mortality**					
Prudent pattern					
No. of deaths	317	283	242	254	
Age-, sex- and area-adjusted HR^b^ (95%CI)	1.00 (ref)	0.89 (0.76 to 1.05)	0.67 (0.57 to 0.79)	0.59 (0.50 to 0.70)	<0.001
Multivariate-adjusted HR^c^ (95% CI)	1.00 (ref)	0.94 (0.80 to 1.11)	0.72 (0.61 to 0.85)	0.63 (0.53 to 0.75)	<0.001
Westernized pattern					
No. of deaths	431	279	213	173	
Age-, sex- and area-adjusted HR^b^ (95%CI)	1.00 (ref)	0.82 (0.71 to 0.96)	0.77 (0.65 to 0.92)	0.85 (0.70 to 1.03)	0.016
Multivariate-adjusted HR^c^ (95% CI)	1.00 (ref)	0.86 (0.73 to 0.996)	0.81 (0.68 to 0.96)	0.88 (0.73 to 1.07)	0.06
Traditional Japanese pattern					
No. of deaths	274	253	221	348	
Age-, sex- and area-adjusted HR^b^ (95%CI)	1.00 (ref)	0.88 (0.73 to 1.06)	0.69 (0.57 to 0.84)	0.91 (0.75 to 1.10)	0.27
Multivariate-adjusted HR^c^ (95% CI)	1.00 (ref)	0.91 (0.76 to 1.10)	0.72 (0.59 to 0.88)	0.92 (0.76 to 1.11)	0.27

Abbreviations: CI, confidence interval; HR, hazard ratio.

^a^Based on Cox proportional hazards model, assigning ordinal numbers 0–3 to the quartile of each dietary pattern score.

^b^Adjusted for age (year), sex, and study area (11 areas).

^c^Additionally adjusted for body mass index (<21, 21–22.9, 23–24.9, 25–26.9, or ≥27 kg/m^2^), smoking status (never, past, current with a consumption of <20 or ≥20 cigarettes/day), total physical activity (quartile of metabolic equivalent task-hour/day), history of diabetes mellitus (yes or no), history of hypertension (yes or no), and total energy intake (kcal/day).

In stratified analyses by BMI, hypertension, sex, or age group, significant interactions were observed only for the Westernized dietary pattern (Table 4). Specifically, the interactions by BMI or hypertension were statistically significant for all-cause and CVD mortality; the inverse associations with all-cause and CVD mortality were observed in non-obese participants (BMI <25 kg/m^2^) and those without hypertension but not in obese participants (BMI ≥25 kg/m^2^) and those with hypertension. Significant interactions by sex were observed for all-cause and cancer mortality; the inverse associations with all-cause and cancer mortality were observed in men but not in women. Additionally, significant interactions by age were observed for CVD mortality; the dietary pattern was inversely associated with CVD mortality in older participants only (≥60 years old).

**Table 4:** Multivariable-adjusted^a^ hazard ratios (95% CI) for all-cause, cancer, and cardiovascular disease mortality according to quartile (Q) of the Westernized dietary pattern score by body mass index, history of hypertension, sex, and age.

	Q1 (low)	Q2	Q3	Q4 (high)	*P* trend^b^	*P* interaction
**Stratified by BMI**^c^						
All-cause mortality						
BMI <25 kg/m^2^	1.00 (ref)	0.94 (0.89 to 0.997)	0.89 (0.83 to 0.95)	0.89 (0.83 to 0.96)	<0.001	0.004
BMI ≥25 kg/m^2^	1.00 (ref)	0.94 (0.85 to 1.05)	0.92 (0.83 to 1.03)	0.99 (0.88 to 1.12)	0.68	
Cancer mortality						
BMI <25 kg/m^2^	1.00 (ref)	0.95 (0.87 to 1.04)	0.86 (0.77 to 0.95)	0.90 (0.80 to 1.002)	0.009	0.20
BMI ≥25 kg/m^2^	1.00 (ref)	0.81 (0.69 to 0.95)	0.93 (0.79 to 1.10)	0.98 (0.82 to 1.17)	0.97	
CVD mortality						
BMI <25 kg/m^2^	1.00 (ref)	0.98 (0.88 to 1.10)	0.85 (0.75 to 0.97)	0.82 (0.71 to 0.96)	0.002	0.004
BMI ≥25 kg/m^2^	1.00 (ref)	1.01 (0.83 to 1.24)	0.89 (0.72 to 1.11)	1.09 (0.86 to 1.37)	0.83	
**History of hypertension**^d^						
All-cause mortality						
Yes	1.00 (ref)	0.99 (0.91 to 1.08)	0.97(0.88 to 1.07)	0.99 (0.88 to 1.10)	0.67	0.01
No	1.00 (ref)	0.91 (0.86 to 0.97)	0.85(0.80 to 0.90)	0.88 (0.82 to 0.94)	<0.001	
Cancer mortality						
Yes	1.00 (ref)	1.06 (0.91 to 1.23)	1.05 (0.88 to 1.24)	0.98 (0.80 to 1.19)	0.98	0.12
No	1.00 (ref)	0.86 (0.79 to 0.94)	0.82 (0.74 to 0.90)	0.89 (0.80 to 0.99)	0.005	
CVD mortality						
Yes	1.00 (ref)	0.98 (0.84 to 1.15)	0.95 (0.80 to 1.14)	1.03 (0.84 to 1.26)	0.99	0.03
No	1.00 (ref)	0.97 (0.86 to 1.09)	0.79 (0.69 to 0.91)	0.81 (0.70 to 0.94)	<0.001	
**Stratified by sex**^e^						
All-cause mortality						
Men	1.00 (ref)	0.92 (0.87 to 0.98)	0.86 (0.81 to 0.92)	0.89 (0.82 to 0.95)	<0.001	0.005
Women	1.00 (ref)	0.96 (0.89 to 1.04)	0.93 (0.85 to 1.01)	0.96 (0.87 to 1.06)	0.20	
Cancer mortality						
Men	1.00 (ref)	0.89 (0.81 to 0.98)	0.82 (0.74 to 0.91)	0.91 (0.81 to 1.02)	0.015	0.003
Women	1.00 (ref)	0.95 (0.83 to 1.08)	0.97 (0.85 to 1.12)	0.94 (0.80 to 1.10)	0.48	
CVD mortality						
Men	1.00 (ref)	0.96 (0.85 to 1.08)	0.81 (0.71 to 0.94)	0.83 (0.71 to 0.97)	0.003	0.13
Women	1.00 (ref)	1.00 (0.87 to 1.16)	0.90 (0.76 to 1.06)	0.95 (0.78 to 1.15)	0.34	
**Stratified by age**						
All-cause mortality						
<60 years old	1.00 (ref)	0.92 (0.84 to 1.01)	0.93 (0.84 to 1.02)	0.93 (0.84 to 1.02)	0.17	0.09
≥60 years old	1.00 (ref)	0.94 (0.89 to 0.995)	0.86 (0.80 to 0.92)	0.89 (0.82 to 0.96)	<0.001	
Cancer mortality						
<60 years old	1.00 (ref)	0.96 (0.83 to 1.10)	0.95 (0.83 to 1.10)	1.02 (0.88 to 1.18)	0.77	0.06
≥60 years old	1.00 (ref)	0.89 (0.81 to 0.98)	0.83 (0.74 to 0.92)	0.85 (0.75 to 0.96)	<0.001	
CVD mortality						
<60 years old	1.00 (ref)	1.01 (0.83 to 1.22)	0.97 (0.80 to 1.19)	0.97 (0.79 to 1.20)	0.71	0.014
≥60 years old	1.00 (ref)	0.97 (0.87 to 1.08)	0.80 (0.70 to 0.91)	0.84 (0.72 to 0.98)	<0.001	

Abbreviations: BMI, body mass index; CVD, cardiovascular disease.

^a^Adjusted for age (year), sex, study area (11 areas), body mass index (<21, 21–22.9, 23–24.9, 25–26.9, or ≥27 kg/m^2^), smoking status (never, past, current with a consumption of <20 or ≥20 cigarettes/day), total physical activity (quartile of metabolic equivalent task-hour/day), history of diabetes mellitus (yes or no), history of hypertension (yes or no), and total energy intake (kcal/day).

^b^Based on Cox proportional hazards model, assigning ordinal numbers 0–3 to the quartile of each dietary pattern score.

^c^Adjusted for the same variables as in footnote a, but BMI was used as a continuous variable.

^d^Adjusted for the same variables as in footnote a with the exception of history of hypertension.

^e^Adjusted for the same variables as in footnote a with the exception of sex.

## Discussion

In this large-scale, population-based, prospective study among Japanese men and women, the prudent dietary pattern, which was characterized by a high intake of vegetables, fruits, soy products, potatoes, seaweed, mushrooms, and fish, albeit a high intake of salt, was significantly associated with a decreased risk of all-cause, CVD, heart disease, and cerebrovascular disease mortality. The Westernized dietary pattern, which was characterized by a high intake of meat, processed meat, bread, dairy products, coffee, black tea, soft drink, dressing, sauce, and mayonnaise but a low intake of salt, was also inversely associated with the risk of all or cause-specific mortality. The traditional Japanese dietary pattern was not associated with the risk of any type of mortality. To our knowledge, this is first study to inclusively examine the association between dietary pattern and all-cause and major cause-specific mortality in Japanese adults.

Our findings of the inverse associations between the prudent dietary pattern and all-cause and CVD mortality is in line with those of a meta-analysis that included 7 studies (6 in Western countries and 1 in Asia) for all-cause mortality and 6 studies (2 in Western countries and 4 in Asia) for CVD mortality, which reported 24% and 19% lower risk of all-cause and CVD mortality, respectively, among those in the highest category of the “prudent/healthy” dietary pattern (high intake of vegetables, fruits, fish, poultry, whole grains, and low-fat dairy products) compared to the lowest category [4]. In addition, Kumagai et al. [5] found an inverse association for a dietary pattern characterized by high intake of soybean products, vegetables, seaweeds, potatoes, and fruit, with all-cause mortality among Japanese elderly (>65 years old). In two other Japanese studies [6,7], a “Japanese” diet pattern (high intake of soybean products, fish, seaweeds, vegetables, fruit, and green tea) and a “vegetable” diet pattern (high intake of vegetables and fruit, mushrooms, seaweeds, and bean curd) were associated with decreased risk of CVD mortality. These previous findings are consistent with our findings. In the present study, participants with higher scores of the prudent dietary pattern consumed greater amounts of polyunsaturated fatty acids, minerals (including potassium and magnesium), vitamins, and fiber, which have been associated with lower risk of CVD, including heart disease and stroke [15–21], compared to those with lower scores. Therefore, the inverse association of the prudent dietary pattern with mortality may be ascribable to the combined effect of these nutrients.

In the present study, the Westernized dietary pattern was also inversely associated with all-cause and CVD mortality. This finding is inconsistent with the findings of previous studies. In the meta-analysis (in which 6 of 7 studies were conducted in Western countries) [4], the “Western/unhealthy” dietary pattern (high intake of processed and/or red meat, refined grains, sweets, desserts, eggs, and high-fat dairy products) was not associated with all-cause or CVD mortality. Of two Japanese studies, one showed an increased risk of CVD mortality associated with an “animal food” pattern (high intake of beef, pork, ham, sausage, chicken, liver, butter, coffee, and alcohol) [7]. In contrast, another study observed no association between an “animal food” pattern (high intake of meats, fishes, and deep-fried foods or tempura) and mortality risk [6]. The consumption of meat (including processed meat), which characterized the Westernized dietary pattern, has been reported to be associated with an increased risk of all-cause mortality in a meta-analysis [22]. However, consumption of meat is low in the Japanese population. According to the Food and Agriculture Organization of the United Nations, Japanese consume 48.8 kg of meat per capita, whereas Americans consume 117.6 kg of meat per capita [23]. Moreover, the potentially unfavourable effect of meat (including processed meat) on mortality might be negated by a beneficial effect of other foods, such as coffee [24,25] and milk and dairy products [26], foods characterizing the Westernized pattern in the present study. Participants with a higher score of the Westernized dietary pattern consumed a greater amount of saturated fatty acids than those with a lower score. Given the inverse association of saturated fatty acids and CVD risk in the JPHC study population [27], saturated fatty acids may also contribute to the inverse association with the Westernized dietary pattern. Low intake of salt, which was associated with the Westernized pattern, has been associated with lower risk of CVD [28].

Our finding of an inverse association between the Westernized dietary pattern and cancer mortality is also inconsistent with previous findings. In the Nurses’ Health Study [29], a “Western” dietary pattern (high intakes of red meat, processed meat, refined grains, French fries, and sweets/desserts) was associated with a higher risk of cancer mortality. In the Singapore Chinese Health Study [30], a “dim sum- and meat-rich” dietary pattern (high intake of dim sum, fresh and processed meats and seafood, noodle and rice dishes, sweetened foods, and deep-fried foods) was positively associated with cancer mortality in ever smokers. However, in the Whitehall II Study [31], compared with the “unhealthy” cluster (white bread, processed meat, fries, and full-cream milk), the others (“sweet”, “Mediterranean-like”, and “healthy” clusters) were not associated with cancer mortality. No Japanese study has examined the association between dietary pattern and overall cancer mortality. It is difficult to compare the association of dietary patterns with overall cancer mortality between the present study and previous studies of non-Japanese because the main causes of cancer differ among countries. In the US and the UK, the major causes of cancer mortality are lung, prostate, breast, and colorectal cancer [32]. On the other hand, the main causes in Japan are lung, stomach, and colorectal cancer [32]. The “western/unhealthy” dietary pattern has been reported to be associated with increased risk of stomach [33], colon [34], and lung cancer [35] in meta-analysis (predominantly including studies in Western countries) and previous studies conducted in Western countries. However, in some [8,36–40] but not all Japanese studies [41], “western”, “meat”, “animal food”, and “high-fat” dietary patterns were not associated with the incidence or mortality of stomach cancer or colorectal cancer and adenomas. According to the World Cancer Research Fund [42] and the National Cancer Center of Japan [43], fruit intake was associated with lung cancer; salt, vegetable, and fruit intake were associated with stomach cancer; and red meat, processed meat, dietary fiber, and calcium intake were associated with colorectal cancer. The Westernized dietary pattern was positively associated with meat intake, but was less strongly associated with intake of vegetables and fruits. In addition, the Westernized dietary pattern was inversely associated with salt intake, but was also inversely associated with intake of calcium and dietary fiber. Given these associations, an increased risk of cancer mortality associated with the Westernized dietary pattern may be expected. The present finding of an inverse association between the Westernized dietary pattern and cancer mortality, as well as the much higher stomach cancer mortality rate in Japan compared to Western countries, might be partly explained by low salt intake in those with higher scores of the Westernized dietary pattern. Alternatively, the inverse association between the Westernized dietary pattern and cancer mortality might be ascribed to the effect of other foods or nutrients and the cancelling or synergistic effects by combinations of several foods or nutrients. Because the association between cancer and dietary patterns may differ by site of cancer and country, further investigation of cause-specific cancer among Japanese is required.

Major strengths of the present study included its large sample size, population-based prospective design, long follow-up period, relatively few participants lost during follow-up, use of a validated FFQ, and adjustment for or stratification by potentially important confounding variables, including hypertension and obesity. Death registration in Japan, which we used to identify the causes of death, is believed to be exhaustive. However, several limitations of the present study warrant mention. First, dietary intake was assessed at only one time point, so long-term habits may not be accurately reflected. Repeated assessment of diet over a long period of time before death would likely provide a better estimate of exposure status. Second, principal component analysis requires subjective decisions in determining the number of factors to retain, choosing the method of rotation of the initial factors, and labelling the dietary patterns. However, we maintain that the validity and reproducibility of the three dietary patterns derived from the subsample was acceptable [14]. Third, we excluded participants who did not respond to both baseline and second surveys. We could not rule out the possibility of selection bias due to these exclusions. Fourth, due to the small number of participants in each subgroup, the results of subgroup analyses may be due to chance and should be interpreted with caution. Fifth, we cannot rule out the possibility of unmeasured and residual confounding, including the effects of socioeconomic status. Finally, the present findings may not be applicable to non-Japanese populations because different ethnic groups or populations may have different dietary patterns.

In conclusion, we found that a prudent dietary pattern, which was characterized by a high intake of vegetables, fruit, soy products, potatoes, seaweed, mushrooms, and fish, albeit a high intake of salt, was associated with a decreased risk of all-cause, CVD, heart disease, and cerebrovascular disease mortality among Japanese adults. In addition, a Westernized dietary pattern, which was characterized by a high intake of meat, processed meat, bread, dairy products, coffee, black tea, soft drink, dressing, sauce, and mayonnaise but a low intake of salt, was inversely associated with all-cause, cancer, CVD, heart disease, and cerebrovascular disease mortality. These findings suggest that the prudent and Westernized dietary patterns are protectively associated with death from all causes, especially CVD, in Japanese populations. Further investigation is required to confirm the inverse association between the Westernized dietary pattern and mortality, which was observed only in some subgroups.
